# Regulatory T cell and macrophage crosstalk in acute lung injury: future perspectives

**DOI:** 10.1038/s41420-023-01310-7

**Published:** 2023-01-16

**Authors:** Tianshu Guan, Xv Zhou, Wenwen Zhou, Hui Lin

**Affiliations:** 1 Department of Pathophysiology, School of Basic Medical Sciences, Nanchang University, 330006 Nanchang, Jiangxi China; 2 Queen Mary university, Nanchang University, 330006 Nanchang, Jiangxi Province China

**Keywords:** Respiratory tract diseases, Adaptive immunity

## Abstract

Acute lung injury (ALI) describes the injury to endothelial cells in the lungs and associated vessels due to various factors. Furthermore, ALI accompanied by inflammation and thrombosis has been reported as a common complication of SARS-COV-2 infection. It is widely accepted that inflammation and the cytokine storm are main causes of ALI. Two classical anti-inflammatory cell types, regulatory T cells (Tregs) and M2 macrophages, are theoretically capable of resisting uncontrolled inflammation. Recent studies have indicated possible crosstalk between Tregs and macrophages involving their mutual activation. In this review, we discuss the current findings related to ALI pathogenesis and the role of Tregs and macrophages. In particular, we review the molecular mechanisms underlying the crosstalk between Tregs and macrophages in ALI pathogenesis. Understanding the role of Tregs and macrophages will provide the potential targets for treating ALI.

## Facts

- Regulatory T cells (Tregs) and macrophages are responsible for acute lung injury (ALI) progression through pro-/anti-inflammatory cell balance.
- Tregs and macrophages influence each other to potentiate their roles in ALI.
- Based on the mutual interaction, new application of conventional drugs and novel insights of ALI treatment are generated.

## Open Questions

- The specific Treg–macrophage crosslink in different stage of ALI needs more investigations.
- The curative effects of application of conventional drugs and new immunological therapy should be performed in precise clinical verifications.
- The proper control of Treg or M2 macrophage expansion in ALI treatment expects further studies.

## Introduction

Acute lung injury (ALI) is a common pulmonary injury characterized by dyspnea and cyanosis due to insufficient oxygenation [1]. Diffuse alveolar damage (DAD) is one of the most common manifestations of ALI, as well as in COVID-19 [2, 3]. From histological observation, it can be divided into three stages according to the pathological process: the acute or exudative stage, the organizing or proliferative stage and the late or fibrotic stage. The first stage involves an acute inflammatory response. Increased blood flow and vascular permeability cause ischemia and even hemorrhage of alveolar vessels. Diapedesis and chemotaxis of blood cells, as well as the transcytosis of proteins, can decrease the colloid osmotic pressure of blood, resulting in edema [4]. Emigrating cells and proteins begin to form a hyaline membrane, which is a characteristic of this stage [5, 6]. After that the membrane grows and develops into granulation tissue in the organizing stage, due to the proliferation of fibroblasts in the pulmonary interstitium [7]. In the last stage, fibrosis of granulation tissue is accompanied by deposition of extracellular matrix proteins. Then, reorganization of connective tissue and vessels leads to the scar formation [7–9]. Nowadays ALI is widely regarded to associate with COVID-19. In the disease, SARS-COV-2 infects the lung by attaching to angiotensin-converting enzyme and causes a series of related symptoms, including alveolar injury, abnormal vasculature, and pulmonary edema, as verified through postmortem examination [10].

At the cellular level, several immune cells play a role throughout the pathological process of ALI. Macrophages and regulatory T cells (Tregs) are two typical immune cell types. As the first cell to detect stimuli, macrophages are essential for the initiation of immune cascades, which can either contribute to or inhibit the development of ALI [11, 12]. In addition, Tregs, which attenuate excessive responses, have been reported to prevent the progression of the disease by acting on type II alveolar cells [13, 14]. These findings confirm the involvement of these two cell types in ALI pathogenesis. Therefore, it can also be assumed that they mutually interact with each other. In this review, we briefly discuss the roles of macrophages and Tregs in ALI. Furthermore, we discuss and summarize the crosstalk between macrophages and Tregs in ALI and potential clinical treatments related to their interaction, including new therapies in COVID-19.

## Tregs and their function in ALI

Tregs are specific T cells that work for immunosuppression to prevent self-reactivity. According to their origin, they can be divided into natural Tregs (nTregs) and induced Tregs (iTregs), which are derived from the thymus and naïve T helper cells, respectively [15].

nTregs are more common. They possess more regulatory functions than iTregs and express CD4 + CD25 + Foxp3+ surface biomarkers [16, 17]. For production, CD4 + T cells receive a relatively weak T cell receptor (TCR) signal, allowing the generation of CD25 and Foxp3. Then, specific cytokines, such as IL-2, stimulate the maturation of nTregs [18, 19]. nTreg differentiation is also controlled by other factors, like TGF-β. Recent findings have revealed that TGF-β helps the oriented differentiation to Tregs but not effector T cells, through regulation of several transcription factors [19–22]. Foxp3 gene is critical and characteristic for the function of nTregs because it acts as a regulator of Treg immunosuppression by upregulating the expression of other surface molecules, including CD25 and CTLA-4. It also aids in inhibiting the transcription of several key immune factors, such as IFN-γ [19, 23, 24]. Several clinical trials suggest that patients with Foxp3 mutations are more likely to develop inflammatory disorders, while restoration of Foxp3 function can compensate for the loss of Foxp3 function [25–27].

iTregs play a relatively small role in ALI. They have two main types, Tr1 and Th3 cells (both are Foxp3−). Rather than Foxp3, CD49b and LAG-3 are characteristic biomarkers of Tr1 cells [28]. Tr1 cells perform their function mainly by secreting IL-10 or TGF-β, both of which are potent inhibitors of inflammation [28–30]. Research on Th3 cells starts from oral tolerance [31, 32]. Current investigations are still relatively limited. However, some evidence indicates that LAP is a Th3 cell biomarker, induced by TGF-β [33]. Another distinct type of iTregs resemble Tregs, with CD4, CD25 and Foxp3, but they are still generated from peripheral CD4 + T cells [34]. Their reproduction is often induced by TGF-β and IL-2 in response to normal stimulation, such as the binding of microbiome metabolites or food antigens to Toll-like receptors (TLRs) [34–36].

In ALI, Tregs often cooperate with Th17 cells, and a proper Th17/Treg balance is important for this collaboration. When STAT3-RORγt dominates in CD4 + T cells, more Th17 cells are differentiated and inflammation will be promoted [37, 38]. In this condition, the level of IL-17 secreted by Th17 cells is higher than the level of IL-35, a marker of Tregs [39]. IL-17 binds to specific receptors on the alveolar epithelium, resulting in the release of chemokines such as CXCL8. Neutrophils respond to these cytokines and are recruited to the injury site [40]. In addition, IL-17 acts directly on monocytes to facilitate their maturation and extravasation, leading to macrophage recruitment [41]. As a result, several immune cells infiltrate into the lesion. IL-17 also assists in oxidative free radical production, which causes further damage to alveolar epithelial cells and microvessel endothelial cells [42]. Finally, detrimental changes that occur in the exudative phase of ALI, i.e., increased blood flow, immune cell infiltration and edema, are exacerbated [43]. In contrast, if the Th17/Treg ratio is decreased due to higher transcription of STAT5-Foxp3, multifaceted compensatory mechanisms are initiated [37, 38]. First, Tregs work against Th17 cells by producing IL-13 when IL-33 is released from the injured epithelium. IL-13 can inhibit the inflammatory effects of monocytes by preventing macrophage differentiation and infiltration [44]. In addition, Tregs inhibit neutrophil function and promote tissue regeneration via a vital mechanism. They secrete TGF-β, which induces neutrophil apoptosis to decrease the number of neutrophils and create a favorable environment for tissue repair [45–47]. Tregs also directly activate type II alveolar cells, leading to their proliferation and differentiation into type I alveolar cells to achieve regeneration of the alveolar epithelium [13].

When the Th17/Treg ratio is balanced, Tregs are also modulated by other mediators, such as an important enzyme, protein kinase B (PKB/Akt). This enzyme has a dual effect in ALI. In the early stage of ALI, infection decreases Akt phosphorylation in pulmonary vascular endothelial cells, leading to elevation of FoxO1/3a level. The increase in FoxO1/3a expression diminishes cell‒cell junction protein level, leading to leakage of pulmonary vessels and edema, which aggravate ALI [48]. However, inactivation of Akt elevates Foxp3 expression and the number of Tregs bursts. Finally, Tregs perform their function to inhibit neutrophil infiltration and achieve alveolar injury resolution to ameliorate ALI [49]. Therefore, inhibitors of Akt, such as phosphatase and tensin homolog deleted on chromosome 10 (PTEN), effectively ameliorate ALI by activating Tregs in the final stage [50].

**Fig. 1:**
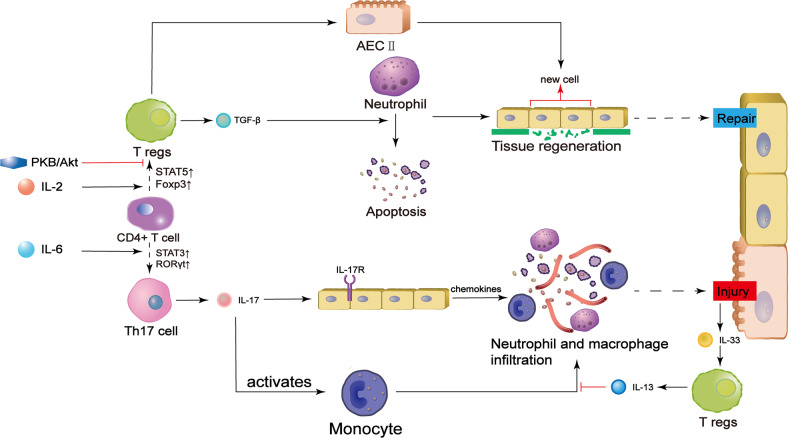
Role of T cells in ALI. Under the effects of various cytokines, CD4 + T cells differentiate into different cell types. IL-2 initiates the transcription of STAT5 and Foxp3 to induce Treg conversion, while IL-6 is necessary for the activation of Th17 cell-specific transcription factors. In addition, PKB/Akt acts intracellularly to prevent Treg differentiation. Tregs secrete TGF-β, inducing neutrophil death or preventing neutrophil extravasation. Tregs also stimulate the proliferation and differentiation of type II alveolar cells (AECII) to achieve direct repair of the alveolar epithelium. In contrast, Th17 cells produce IL-17 and exacerbate inflammation by inducing neutrophil infiltration and monocyte maturation. The role of monocytes in ALI is also inhibited by Tregs. IL interleukin, TGF tissue growth factor, STAT signal transducer and activator of transcription, RORγt retinoid-related orphan nuclear receptor γt, Foxp3 forkhead box protein 3.

In short, Tregs play a significant role in alleviating self-reactivity in ALI by regulating immune reactions and preventing autoimmune disorders, and in vivo cellular factors and drugs that impact these cells can be used to treat ALI. Th17/Treg balance is dominant in the process, which is influenced by extracellular cytokines and intracellular enzymes [37, 38, 48]. Th17 cells, which are proinflammatory, produce IL-17 to activate immune cells through transcriptional regulation, ultimately inducing excessive recruitment of immune cells and lung injury [51]. In contrast, Tregs secrete TGF-β and subsequently promote resolution either directly or indirectly through neutrophil deregulation or type II alveolar cell stimulation [13, 45, 47] (Fig. 1).

## Macrophages and their function in ALI

Macrophages are myeloid cells that are essential for innate immunity. According to their location in lung, they are classified to two types, i.e., alveolar macrophage and interstitial macrophage [52]. The former is present on the inner surface of alveoli and exposed to the outer environment directly, playing a major role in the first line of defense in immune reactions [53]. The latter is often associated with airway, nerves and vessels, but there is still lack of relevant research [54–56]. Originally, monocytes circulate in the blood, where they mature and undergo chemotaxis [49]. Upon recognition of damage-associated molecular patterns (DAMPs) or pathogen-associated molecular patterns (PAMPs) by TLRs/NOD-like receptors (NLRs), macrophages become polarized and produce several cytokines, which perform different functions in inflammation [57, 58]. Macrophages are polarized towards the M1 or M2 phenotype under different conditions, and M1 and M2 macrophages produce different cytokines [59]. The polarization of M1 macrophages, which express the surface molecules CD80, CD86 and CD16/32, can be triggered by IFN-γ or lipopolysaccharide, which promote antigen elimination. Once activated, M1 macrophages secrete proinflammatory cytokines such as TNF-α or IL-6 [60]. In terms of inflammation energetics, M1 macrophages increase glycolysis and promote the pentose phosphate pathway, resulting in rapid production of ATP for immune reactions [61, 62]. Considering inflammatory mediators, promotion of the pentose phosphate pathway also results in the production of more NADPH, which facilitates the production of nitrogen intermediates and ROS [63]. This process also inhibits the tricarboxylic acid (TCA) cycle by downregulating the expression of isocitrate dehydrogenase and increasing the level of lipid metabolites such as leukotriene and IL-1β, which are also important pro-inflammatory factors [64]. M2 macrophages, which are activated by IL-4 and IL-13, are responsible for inflammation resistance and tissue repair [60]. M2 macrophages produce anti-inflammatory cytokines, such as TGF-β or IL-10, which can also induce Treg differentiation. M2 macrophages depend more on persistent energy generation by oxidative phosphorylation and fatty acid oxidation, which are important for inflammation resolution [65]. Unlike M1 macrophages, TCA cycle is intact in M2 macrophages, preventing the accumulation of TCA intermediates and the overexpression of proinflammatory factors [64, 66].

Differentiation of macrophages depends on the progression of ALI. In the early acute phase, upon recognition of microbial products, M1 macrophages are activated to initiate inflammation by secreting abundant inflammatory substances, including IL-1 and TNFα [67]. These two cytokines help to recruit neutrophils to the lesion through MyD88 and NF-κB activation [68, 69]. Influx of neutrophils potentiates antigen elimination and self-protection, but these cells also attack normal tissue. For example, they secrete many types of proteinases and ROS, which injure alveolar epithelial cells and reduce the levels of surface proteins. Both of them are important for gas exchange [70]. They also cause blood vessel damages by impairing vessel permeability. Therefore, more fluid flows into the alveoli and lung interstitium and leads to edema [71, 72]. Polarization towards the proinflammatory M1 phenotype is regulated by many factors. Studies have demonstrated the important roles of suppressor of cytokine signaling 3 (SOCS3), NF-κB and so on in this process [70, 73]. In addition, c-Jun N-terminal kinase (JNK) is interesting because it has a dual effect on the polarization of macrophages. While JNK can promote the development of ALI along with the activation of p38 mitogen-activated protein kinase (MAPK), the expression of JNK and transcription of c-Myc are important for M2 polarization [74, 75]. In the organizing and fibrotic stages, M2 macrophages dominate. M2 macrophages influence injury resolution in two ways. First, M2 macrophages promote alveolar fibrosis by secreting specific cytokines, such as IL-1 and TGF-β. IL-1 attracts fibrocytes, while TGF-β induces the conversion of fibroblasts to myofibroblasts. M2 macrophages also produce and cause the deposition of collagen, resulting in the formation of fibers that cover the lesion on the alveolar epithelium [76–78]. Second, M2 macrophages induce alveolar regeneration directly. They can act on type II alveolar cells to promote type I cell differentiation and surfactant secretion [79]. Overall, both M2 and M1 macrophages are essential for the progression of ALI.

**Fig. 2:**
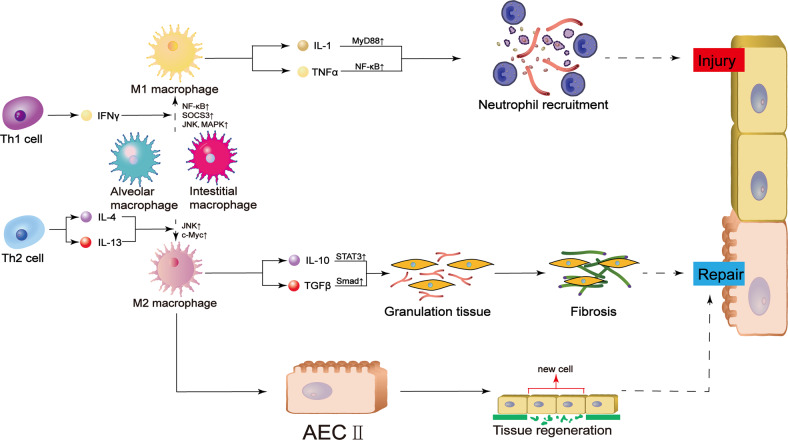
Role of macrophages in ALI. The progression of ALI is influenced by alveolar and interstitial macrophage polarization. T helper 1-mediated production of IFNγ induces M1 polarization, with increased transcription of NF-κB, SOCS3, JNK and MAPK. M1 macrophages promote neutrophil recruitment either through phagocytosis and subsequent cell damage or through cytokine secretion and transcription factor activation. IL-4 and IL-13 polarize alveolar macrophages towards the M2 phenotype by regulating the transcription of JNK and c-Myc. M2 macrophages secrete cytokines that induce fibrosis or stimulate new cell regeneration. Both processes promote tissue repair. IFN interferon, TNF tissue necrosis factor, MyD88 myeloid differentiation primary response 88, NF-κB nuclear factor-kappaB, JNK c-Jun N-terminal kinase, AECII type II alveolar epithelial cell.

Therefore, alveolar and interstitial macrophages are tightly controlled during ALI progression and recovery. First, cytokines produced by T helper 1 cells, such as IFN-γ, aid the polarization of macrophages towards the M1 phenotype, and M1 macrophages secrete cytokines, such as IL-1 and TNFα, for transcriptional regulation [68]. These two cytokines promote neutrophil recruitment and cause damage to alveoli and pulmonary microvessels [69]. When recovery is initiated, anti-inflammatory functions are induced. T helper 2 cells participate in this process and secrete IL-4 and IL-13. They promote the polarization of macrophages towards the M2 phenotype [60]. M2 macrophages can secrete TGFβ and IL-10. These two cytokines promote granulation tissue formation and subsequent fibrosis by acting on fibrocytes and fibroblasts on the alveolar wall. On the other hand, M2 macrophages directly induce type II cell proliferation and tissue regeneration [79]. As a result, cooperation between normal and abnormal repair processes leads to disease recovery (Fig. 2).

## Crosstalk between Tregs and macrophages in ALI

### Extracellular factors act on Tregs to influence cytokine secretion, affecting macrophage cell fate in ALI

As an essential contributor to inflammation inhibition, Tregs are involved in inflammation resolution and repair of lung tissue and therefore prevent ALI progression. Firstly, they prevent disease progression by controlling inflammatory cell fate. For example, IL-33, which is produced by the lung epithelium after injury, could prevent the progression of ALI by promoting the proliferation of Tregs. Specifically, IL-33 binds to ST2 receptor on the surface of Tregs and stimulates secretion of IL-13 by them [44]. One study reported the possible mechanisms by which IL-13 regulates ALI progression. First, Tregs secrete IL-13, which consequently stimulates the production of IL-10 by macrophages. Autocrine release of IL-10 by macrophages induces the activation of Rac1, which is important for cytoskeleton remodeling and cell engulfment. Vav1 is a type of GTP-exchange factor that is essential for normal Rac1 function. It has also been reported that knockout of Vav1 in macrophages diminishes the effect of IL-10, revealing that Vav1 mediates IL-10-induced apoptosis and internalization by regulating Rac1. In conclusion, an IL-13-IL-10-Vav1-Rac1 axis might exist in ALI and may inhibit ALI progression [80]. Secondly, in addition to promoting macrophage elimination, Tregs also influence the direction of macrophage polarization. Studies have found that when Tregs and monocytes are cultured together, the level of proinflammatory cytokines released by M1 macrophages decreases, while the level of some biomarkers of M2 macrophages increases. These changes are regulated by multiple cytokines secreted by Tregs [81]. One major mechanism underlying these changes is the regulation of IL-10 expression. IL-10 expression is correlated with the phosphorylation of GSK3β and PTEN, which are both mediators of macrophage polarization [82, 83]. Evidence shows that Tregs reverse the decrease in pGSK3β, GSK3β and pPTEN levels, while an IL-10 antibody eliminates this effect. Specifically, knockdown of GSK3β blocks PTEN phosphorylation. Therefore, it is concluded that IL-10 produced by Tregs acts on macrophages by promoting the phosphorylation of GSK3β and the subsequent phosphorylation of PTEN, polarizing macrophages towards the M2 phenotype [83]. Tregs also block the transformation of macrophages to the M1 phenotype. During this process, Tregs affect the function of CD8 + T cells and inhibit their production of IFN-γ, which is a modulator of M1 polarization. As a result, the inhibition of sterol regulatory element binding protein 1 (SREBP1) by IFN-γ is relieved, and fatty acid synthesis is restored. Consequently, normal fatty acid oxidation is maintained, and metabolic pathways in M2 macrophages are promoted [84].

The regulation of macrophages by Tregs also needs contribution of several factors. Kynurenine, an amino acid generated by IDO-mediated tryptophan decomposition, activates aryl hydrocarbon receptors (AHRs) on Tregs and stimulates immunosuppression by macrophages [85]. Evidence has shown that upon stimulation, dendritic cells produce IDO, which initiates kynurenine synthesis in these cells [86]. It is also known that specific amino acids induce immunosuppression through the AHR pathway. In recent studies, AHR was knocked out in Tregs, and the results revealed that the number of M2 macrophages decreased, judged from the levels of surface markers. The results demonstrate that IDO-kynurenine-AHR signaling may regulate Treg-mediated macrophage activation [85]. The possible mechanism may involve transcriptional regulation of Akt and p-GSK3β and consequent IL-8 release [87]. Another factor is netrin-1, produced by neuroepithelial cells. It was found to regulate inflammation, including by acting on Tregs, in recent years. The reduction in the netrin-1 level is a characteristic of ALI and further exacerbates the disease [88]. Furthermore, netrin-1 binds to the A2b receptor and increases IL-10 content to induce the polarization of macrophages towards the M2 phenotype, as mentioned above [88]. Studies in mice have also found that administration of netrin-1 rescues tissue necrosis and leukocyte infiltration by activating and increasing the number of Tregs; thus its effect in controlling macrophage polarization may be achieved by increasing the number of Tregs [89].

**Fig. 3:**
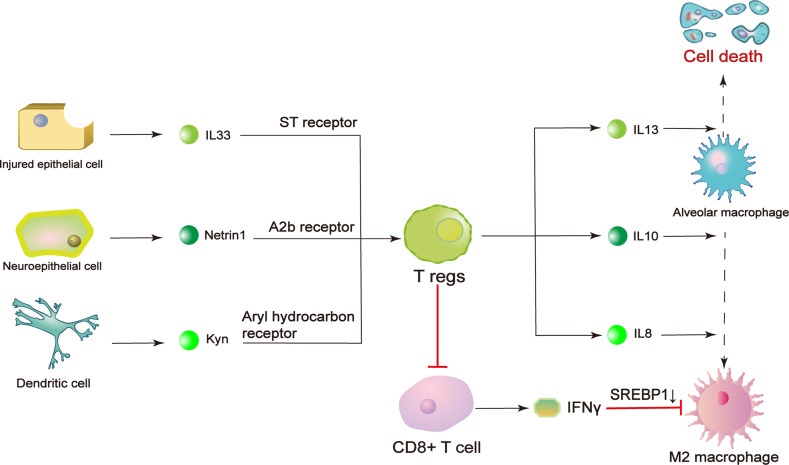
Regulation of macrophage polarization by Tregs. This regulatory effect is controlled by many factors. For example, IL-33 binds to ST receptors on the Treg surface to promote IL-13 secretion, which subsequently induces apoptosis of alveolar macrophages and prevents further inflammation. On the other hand, netrin 1 and kynurenine both promote M2 macrophage polarization. Netrin 1 binds to A2b receptors on Tregs to induce IL-10 secretion. Kynurenine binds to AHRs and modulates transcription in Tregs, increasing the production of IL-8. Furthermore, the production of IFNγ, which inhibits M2 polarization, is inhibited by Tregs. As a result, Tregs can both prevent injury and promote tissue repair by regulating macrophage function. Kyn kynurenine, IFNγ interferon γ, IL interleukin, SREBP1 sterol regulatory element binding protein 1.

In conclusion, several factors are responsible for the regulatory effect of Tregs on macrophage polarization by binding to specific receptors on Tregs. After binding, transcriptional alterations in Tregs lead to the secretion of cytokines, which act on alveolar macrophages to cause cell death or M2 phenotype transformation [80, 83, 87]. Besides, through another pathway that regulates the direction of polarization, Tregs suppress the ability of CD8 + T cells to secrete IFNγ, which is an inhibitor of M2 polarization [84]. When the number of M2 macrophages increases, pulmonary repair is strongly affected. On the one hand, cytokines induce myofibroblast activation [76]. On the other hand, type II alveolar cell stimulation leads to epithelial regeneration [79]. In addition to type II alveolar cells, M2 macrophages are also responsible for endothelial cell restoration and pulmonary angiogenesis. Through regulation of ion channels, excessive fluid in alveoli and interstitium is released to relieve edema of the lungs [90] (Fig. 3).

### Th17/Treg balance is regulated by many factors secreted directly or controlled by M2 macrophages in ALI

Conversely, macrophages also alter the activation of Tregs by releasing special proteins. Two of them are TGFβ and retinal dehydrogenase (RALDH). High level of TGF-β has been detected in lung tissue macrophages, and its antibody can block Foxp3 expression in T cells [91]. Although this finding suggests that TGF-β promotes Treg differentiation, the detailed mechanism remains to be explored. It is currently known that TGF-β promotes Foxp3 expression by preventing the translocation of DNA methyltransferase I, which methylates the Foxp3 gene and downregulates its expression under normal conditions. Moreover, TGF-β targets conserved noncoding sequence-1 (CNS1), an enhancer of the Foxp3 gene, to elevate Foxp3 gene expression [91, 92]. RALDH is now considered as a synergist of TGF-β [91]. It is responsible for the synthesis of retinoic acid, which also acts on CNS1 by binding to related receptors. Retinoic acid has other anti-inflammatory roles. For example, it decreases RORγt level to control the differentiation of CD4 + T cells or increases the production of arginase 1 from dendritic cells. Both mechanisms ultimately promote Treg generation [91, 93–96]. Another pivotal protein is maresin1, which is the oxidative metabolite of docosahexaenoic acid (DHA), in macrophages. It regulates M2 macrophage polarization in ALI by relieving the downregulation of PPARγ [97]. However, a recent study proposed a new function for maresin1. The results showed that maresin1 treatment decreased the Th17/Treg ratio in ALI and increased the levels of anti-inflammatory cytokines [98]. Based on these findings, it can be assumed that maresin1 also regulates the differentiation of naïve T helper cells into Tregs and then promotes M2 macrophage polarization.

Tregs can also have the opposite regulatory effect on macrophages. The most typical example is high-mobility group box-1 protein (HMGB1), a significant contributor to inflammation secreted by macrophages [99]. Experiments in animal models have proved that HMGB1 exerts lethal effects by increasing proinflammatory cytokine content and pulmonary epithelium permeability to allow inflammatory cell infiltration [100]. HMGB1 not only directly interferes with the expression of Foxp3 and CTLA-4 on the Treg surface or avoids the release of Treg-mediated cytokines [100], but also acts on macrophage and promotes inhibiting its function on Treg differentiation. Loss of HMGB1 causes inactivation of phosphatase and tensin homolog (PTEN) and recruitment of β-catenin to the nuclei of macrophages, initiating a kinase cascade in which activated PI3K phosphorylates PDK1 and subsequently Akt to promote TGF-β release [101]. This process ultimately leads to the production of more Tregs. The HMGB1-PTEN axis is considered as an important pathway for immunosuppression.

**Fig. 4:**
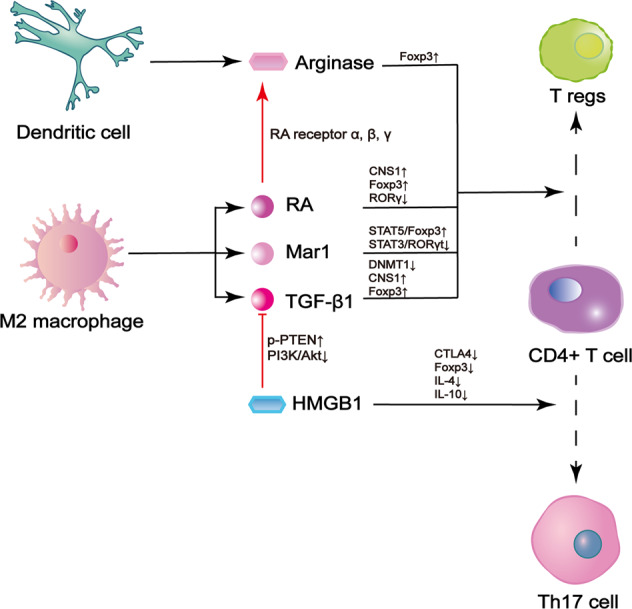
Regulation of T cell differentiation by M2 macrophages. M2 macrophages produce various factors and modulate the transcription of genes, including Foxp3, whose expression is increased, and RORγt, whose expression is decreased. These alterations ultimately lead to Treg differentiation. One important factor, retinoic acid (RA), also activates arginase in dendritic cells and achieves synergism. Other inhibitory factors include HMGB1, which prevents Treg polarization by interfering with induction by M2 macrophage or directly decreases the levels of Treg-related transcription factors. HMGB1 high-mobility group box-1 protein, Akt/PKB protein kinase B, Mar1 maresin 1, DNMT DNA methyltransferase, CTLA4 cytotoxic T-lymphocyte-associated protein 4, CNS1 conserved noncoding sequence-1, RA retinoic acid, TGF tissue growth factor, p-PTEN phosphorylated phosphatase and tensin homolog deleted on chromosome 10, PI3K phosphatidylinositol 3-kinase.

In summary, M2 macrophages determine the fate of CD4 + T cells directly through molecular signaling or indirectly through regulating the function of dendritic cells. However, the common mechanism among all related pathways is upregulation of RORγ and Foxp3, which is important for Treg differentiation [98]. As a result, Tregs initiate alveolar epithelium regeneration by activating proliferative type II alveolar cells. Tregs also inhibit pulmonary damages caused by neutrophils, creating a suitable niche for resolution [13, 45]. A recent study also indicated that Tregs repaired the pulmonary microcirculation through endothelial cell restoration [102]. Conversely, other factors like HMGB1 repress the anti-inflammatory effects of M2 macrophages and induce the production of more Th17 cells. This cell type attracts neutrophils to aggravate epithelial and endothelial injury in the lungs [103, 104]. The shift in the Th17/Treg balance is dependent on the stage of ALI (Fig. 4).

### Treatments for ALI related to Treg-macrophage crosstalk

The prevention and treatment of ALI, a common complication in clinic, is a focus of the medical community. Recently, some new drugs have been studied, with a deeper demonstration about the relationship between the two cells:

1. An antibiotic, *Houttuynia cordata* polysaccharides (HCP), can protect the lung from acute inflammation or injury by affecting cell production, proliferation and migration from Peyer’s patch to the lung. It alters the transcriptional factors in CD4 + T helper cells to control its differentiation orientation. STAT3-RORγt is inhibited, while STAT5-Foxp3 is elevated. More Tregs are produced. The antibiotic also helps the specific migration of Tregs in gut mucosa to lung [105].
2. Preclinical experiments have proven the effectiveness of some drugs in ALI treatment, especially via regulation of Treg-macrophage crosstalk. For instance, luteolin, curcumin, and progranulin all could promote Treg differentiation and further control macrophage polarization [106–108]. In ALI mouse models, administration of these drugs increased the percentage of Tregs and the level of IL-10, resulting in domination of M2 macrophages. As a result, diffused lung inflammation in ALI was alleviated, indicating that these three anti-inflammatory drugs exerted therapeutic effects by ameliorating cell infiltration and cytokine secretion [106–108].
3. Another potential therapeutic target is maresin1, which has a dual effect on cell crosstalk. On the one hand, maresin1 prevents PPARγ inhibition caused by infection and induces M2 macrophage polarization, which might be beneficial for Treg production [97]. Moreover, maresin1 can promote Foxp3 expression while inhibiting RORγt expression in CD4 + T cells [98]. In this case, transcriptional regulation promotes Treg differentiation, which is affected by M2 polarization. In fact, studies have also shown that maresin1 application can successfully alleviate lung inflammation in ALI [98].

## Discussion

ALI, which results from exacerbation of lung inflammation, is the major cause of clinical respiratory failure. Although Tregs and M2 macrophages both exist in its pathogenesis, several factors hinder their function. For example, during ALI, there are several micro vesicles in lung epithelium, which contain specific caspases or miRNAs. They are responsible for stimulating pro-inflammatory M1 macrophages, not M2 type [109, 110]. Considering T cell differentiation, once stimulated by pathogens or inflammatory cytokines, macrophages would release HMGB1. This protein can not only act on macrophage itself and prevent its assistance in Treg differentiation, but also directly impair Treg induction [101, 111]. Moreover, recent study has found a much higher IL-17 level than Foxp3, revealing a serious imbalance between Th17 and Treg [39, 112, 113]. Therefore, it is confirmed that pro-inflammatory Th17 cells still dominate despite of Treg existence. In all, in most time of ALI, neither M2 macrophages nor Tregs could burst.

ALI is also a cause of death in COVID-19, indicating its seriousness. Because our understanding of ALI pathogenesis is limited and poor hemoperfusion accompanied by insufficient tissue oxygenation is the most fatal consequence of this disease, current treatments aim to restore oxygen delivery to the vascular system [114, 115]. For example, the conventional treatment for COVID-19 involves increasing respiratory efficiency, including improving ventilation and promoting vasodilation of pulmonary micro vessels in lesions [116, 117]. With further research, some new treatment methods target the underlying inflammatory process. The most common of them for COVID-19-induced ALI is the administration of corticosteroids, such as dexamethasone, which has been proven to decrease interleukin release and neutrophil activation [118, 119]. Recently, the role of Tregs and macrophages and appropriate therapies have been explored. On the one hand, Th17/Treg balance also exists in COVID-19 infection, modulating the disease. Th17-type reaction is initiated by IL-17. Once it is released from Th17 cell, it attracts neutrophils and macrophages and starts a pro-inflammatory cytokine cascade [44, 120]. On the other hand, studies have revealed the significance of Treg immunosuppression in COVID-19 cases [121]. About macrophages, postmortem examination found that the cells that were invaded by SARS-COV-2 virus expressed a high level of IL-6 [121]. IL-6 cooperates with TGF-β to induce differentiation of CD4 + T cells to Th17 type, instead of Tregs [122, 123]. Other discoveries reveal the possible mechanism of COVID-19 pathogenesis involving Tregs and macrophages. A recent study has found that in COVID-19-induced ALI, Tregs express more Notch4, which is an inhibitor of tissue repair-related cytokines and proteins [124]. In addition, after infected by SARS-COV-2, over-activated JAK-STAT pathway would promote infiltration of inflammatory macrophages and suppress recruitment of Tregs [125]. In the new epidemic, some novel immunotherapies targeting Treg/macrophage might be more appropriate than conventional drug therapy.

Stem cell transplantation is a new thinking for Treg or M2 macrophage restoration. The mechanism is to harness the pluripotency of stem cells to induce more Tregs or macrophages. Now the role of placenta-derived mesenchymal stem cell (pcMSC) and human pluripotent stem cell (hPSC) has been claimed. They can be used to generate more Tregs and M2 macrophages, respectively [126, 127].

However, the novel therapies also need cautiousness. Treatment of ALI must notice a “balance” between Tregs and M2 macrophages. As a routine in immune reaction, spatial or temporal burst of anti-inflammatory cells are both detrimental. Excessive expansion of Tregs and M2 macrophages induce violent immunosuppression, which is common in cancer. Now it has been known that too many Tregs inhibit IFN-γ and subsequently produce more M2 macrophages [84]. In this case, they can enhance immune evasion, allowing progression of cancer metastasis. Therefore, they also provide the potential for pathogen invasion, with an extremely weak immune elimination. From another aspect, too many induced M2 macrophages would potentiate tissue fibrosis, causing the loss of normal organ function [128]. In terms of time, a long persistence of Tregs or M2 macrophages also counteracts. If Tregs maintain a large number until injury resolution, they would initiate a cascade in macrophages and lead to their apoptosis, so both M1 and M2 macrophages are unable to be generated [80]. The result is that the injured tissue cannot be repaired normally, with a high risk of new pathogen invasion. Another study shows that normal physiological roles of macrophages are deprived when they survive until tissue resolution. What’s more, they also lose their surface markers, which characterize them as M1 or M2 type [129]. That means, long-lived macrophages would become “anergic.”

Unfortunately, there is no definitive way to control immunosuppression accurately, but some factors may provide some evidence. Firstly, IL-18 has the capacity to reduce the ability of Tregs, by decreasing the level of Foxp3 transcriptionally and inhibiting the ubiquitination of the protein. It has proved that the cytokine could impede the anti-inflammatory effects of Tregs in ALI [130]. Therefore, application of human recombinant IL-18 has the potential to reverse the negative aspects of overactive immunosuppression. Moreover, therapy of IL-18 should also notice other factors, which symbolize low immunity. No clear indicators have been suggested, but some proteins like arginase-1 and IDO may be suitable due to their implication of immunosuppression [131]. It is assumed that, if the number of specific proteins is higher than normal, IL-18 should be used earlier to avoid the impact of low immunity, and vice versa. However, because of lack of clinical evidence, an accurate guide of arginase-1/IDO level is still unknown, which needs more investigations.

In another dimension, “anergy” of Tregs and M2 macrophages should also be handled. Research found that IL-10 played an important role in Treg-induced macrophage suicide, so application of IL-10 antibody may be appropriate to avoid the effect [80]. Besides, a protein called signal regulatory protein α (SIRPα) is responsible for alveolar macrophage paralysis. It would exert the function through modulating macrophage microenvironment and subsequent intrinsic gene expression [129, 132–134]. In this way, blockage of SIRPα is necessary to keep ability of macrophages. However, there are also no clear indicators which imply initiation of therapy. IL-10 or SIRPα themselves may act as indicators, but more studies should be planned.

Or in another way, controlling the time and dose of treatment strictly could also avoid low immunity-related side effects. For instance, about pcMSC transplantation, a clinical study showed that if COVID-19 patients received (200 ± 20) × 10^5^ stem cells on Day 0 and Day 4 in a course of treatment, the symptoms associated with ALI was greatly alleviated, including much lower mortality, higher PaO_2_/FiO_2_ ratio and lower lactate dehydrogenase (LDH) and C-reactive protein (CRP) level. All of them indicate that this administration schedule may be suitable [127].

## Conclusion

ALI, a common respiratory complication in clinic, results in the exacerbation of primary diseases such as COVID-19. However, clinicians have not yet identified truly effective measures for preventing ALI. In recent years, studies on the role of immune dysfunction in the pathogenesis of ALI have provided some new insights. Two important participants in ALI are Tregs and macrophages, whose roles in ALI progression and mitigation have been fully elucidated in many studies. In this paper, we summarize findings considering the mutual interaction between these two immune cell types to propose new therapeutic approaches for ALI.

1. Basically, the dominant Tregs in ALI are peripheral CD4 + CD25 + Foxp3+ Tregs. Their major role is to secrete various cytokines that affect related gene transcription in M0 macrophages to induce M2 polarization. M2 macrophages exert anti-inflammatory effects.
2. The Th17/Treg balance is involved in the whole course of ALI. A higher Th17/Treg ratio promotes injury, while tissue repair is often associated with a lower Th17/Treg ratio. Factors produced by M2 macrophages promote the Treg fate, which skews the balance in favor of repair.
3. Some plant extracts or intracellular substances have been proven to exert anti-inflammatory effects in ALI through regulation of the Treg-macrophage interaction. Moreover, new specific immunological methods are being explored and are expected to lead to novel therapeutic strategies to ALI in COVID-19.
4. In treatment targeting ALI, the extent of anti-inflammatory cell expansion is also important to control the degree of immunosuppression. Although current studies are relatively insufficient, we summarize existing results and suggest some possible ways to avoid excessive expansion of Tregs and M2 macrophages.

However, some questions still exist in the field. For example, we just build a general framework about interaction between Tregs and macrophages based on current research, which is not accurate enough. To provide more details about the relationship, more exploration about specific Treg-macrophage crosslink in every stage of ALI is necessary, for a better understanding of immune mechanism underlying the disease. For development of new therapy, on the one hand, the effectiveness and potential side effects of new drugs are still uncertain, so clinical trials in different phases are necessary. In the same way, immunological therapy using stem cells needs more investigations, to define an actually proper therapeutic regime for ALI. On the other hand, the level of Tregs and M2 macrophages should be regulated accurately to avoid adverse consequences which are due to excessive immunosuppression. To achieve this goal, more studies are also required.

Overall, this review includes a comprehensive discussion about the roles of Tregs and macrophages in ALI pathogenesis. We retrospectively review previous research on the potential link between Tregs and macrophages and determine the mode and function of their interaction. This review is of great significance for further exploration of methods to prevent or treat ALI from the aspect of immunosuppression, which will help to reduce the incidence and mortality of ALI in clinic. Crucially, it will also benefit the discovery of drugs for the treatment of lung injury associated with COVID-19.

## Supplementary information

Reproducibility Checklist

## Data Availability

The data that support the findings of this study are available from the corresponding author upon reasonable request.
